# Factors Regulating Stem Cell Biology in Development and Disease

**DOI:** 10.1155/2016/7170642

**Published:** 2015-12-20

**Authors:** Stefan Liebau, Paul Gadue, Kodandaramireddy Nalapareddy, Guido von Figura, Alexander Kleger

**Affiliations:** ^1^Institute of Neuroanatomy & Developmental Biology, Eberhard Karls University Tübingen, 72074 Tübingen, Germany; ^2^Center for Neurosensory Systems (ZFN), Eberhard Karls University Tübingen, 72074 Tübingen, Germany; ^3^Children's Hospital of Philadelphia (CHOP), University of Pennsylvania, 3501 Civic Center Boulevard, Philadelphia, PA 19104, USA; ^4^Division of Experimental Hematology & Cancer Biology, 3333 Burnet Avenue, Cincinnati, OH 45229, USA; ^5^II. Medizinische Klinik und Poliklinik, Klinikum rechts der Isar München, Technische Universität München, 81675 Munich, Germany; ^6^Department of Internal Medicine I, University of Ulm, Albert-Einstein-Allee 11, 89081 Ulm, Germany

Stem cells in general are characterized by their ability to self-renew and to differentiate towards various progeny. Great efforts started to give hope since the first discovery of mammalian pluripotency by Stevens and Little, 1954; the field of stem cell research using pluripotent stem cells (PSC) has reached a state of general interest in both science and medicine. Following an overall fast development of* in vivo *and* in vitro *research, stem cells are nowadays regularly implied in developmental and pathomechanistical studies. Their unique ability to generate all cell types of the respective organism allows for the directed differentiation of* in vitro *cultured PSCs into the desired direction. Subsequently, development and function of this differentiated progeny may be investigated in detail. This specifically accounts for the human system where primary materials as well as* in vivo *studies are strongly limited. In addition, the groundbreaking study describing the generation of PSCs from somatic cells by Takahashi and Yamanaka, served as a lift for unlimited use of individual and patient specific stem cell based investigations. Today, these induced pluripotent stem cells (iPSCs) are amongst others utilized to explicitly study human pathomechanisms in a patient specific setting. Moreover, tissue specific stem cell, for example, serving as a cellular reserve and substitute for decayed cells and degenerated tissue, is discussed as a useful source of human tissue and tissue specific cells. Clearly, these adult stem cells have already been successfully used in therapeutic approaches for decades, such as hematopoietic stem cells in bone marrow transplantations.

Numerous studies exploiting stem cells have already been published describing distinct pathomechanisms in rare and common diseases, giving hope for future therapeutic startups.

Moreover, in the current decade, the first reports of using PSCs in therapeutic approaches have been announced and further upcoming clinical trials in several organ systems are approved and ready to start. Nevertheless, it is still crucial to thoroughly understand underlying cellular mechanisms and molecular pathways implied in developmental and pathological surroundings. In that sense, recent reports suggest that “stem cell factors” fulfill additional functions during the stepwise phases of cell lineage commitment. For instance, during germ layer formation, Oct4 and Tbx3 promote mesodermal as well as endodermal fate and limit neuroectoderm differentiation potential. In contrast, Sox2 enhances neuroectoderm specification while restricting mesoderm and endoderm lineage development. Later, these factors are subsequently involved in tissue development. Second, one hallmark of cancer is the ability to reactivate genetic programs known from early development and stem cells. Interestingly, signalling cascades regulating early patterning in the embryo such as Nodal signalling are again overexpressed during cancer development, and “stem cell factors” can drive dysplasia and tumorigenesis suggesting an intimate link between embryonic cell fate decisions, stem cells, and tumour development.

To that end we have gathered a variety of studies under the umbrella of stem cell development and disease to provide a basis to understand the steps from basic stem cell biology, further going to physiological function during development until disturbances in function leading to pathological symptoms.

On the basis of stem cell biology, M. Nawaz et al. summarize the existing knowledge on stem cell derived extracellular vesicles (EVs). Current knowledge includes that stem cells continuously secrete factors into surrounding compartments serving as autocrine as well as paracrine signal modifiers. Moreover, abundance of EVs has been reported that mimic the phenotypes of the cells from which they originate. They hereby seem to play a role in the exchange of genetic information utilizing persistent bidirectional communication. This implies that EVs could regulate stemness, self-renewal, and differentiation in stem cells and their subpopulations.

A poorly investigated stem cell population is the trophoblast stem cell (TSC), responsible for the generation and function of the fetal placenta. S. J. Arnold et al. thoroughly investigated these TSCs concerning their expression profile* in vivo *and* in vitro*. Using a remarkable set of methods and approaches, they broadly contribute to the knowledge on TSCs and provide a well-defined set of markers and transcriptional signatures of these cells. Moreover, they investigate the time-dependent expression of several genes involved in early differentiation events both on mRNA and protein levels.

In the field of adult mesenchymal stem cells (MSCs), which are still considered as a great hope for a variety of clinical implications, K. C. Elahi et al. briefly discuss both the facts that isolated MSCs are blend of distinct cells and MSCs isolated from different tissues show besides some common features some significant differences. Nevertheless, they point to the fact that these differences could also be a benefit for distinct approaches. For example, subsets of MSCs seem to express distinct and unique sets of surface markers, which might help to find a perfect subpopulation for a respective utilization.

In the system of MSCs, S. Wang et al. provide a study applying transcriptome analyses on Toll-like receptor 3-activated MSCs. TLR3 activity in MSCs was reported to have an impact on MSC function and might therefore be a candidate to understand MSC physiology. Nevertheless, long noncoding RNAs (lncRNAs) were thought to play a role in this system. S. Wang et al. now give a thorough overview on involved lncRNAs using genome-wide transcriptome arrays which might be useful for further studies in TLR3-activated MSCs.

In terms of MSC differentiation signals, J. Chen et al. contribute a study describing the impact of TGFß/Smad signaling for chondrogenic differentiation. Here they explore the role of exogenous heparin sulfate (HS) in terms of its underlying molecular mechanism in MSC chondrogenesis. They applied HS both alone and in combination with TGF-ß and thereby modified TGF-ß receptor expression and ratios, also having an impact on Smad 2/3 activity.

In the neural system, enteric clusters of neurons are crucial to uphold gastrointestinal movement and regulation. Here, P. H. Neckel et al. provide new information on the transcriptome of enteric neural system (ENS) progenitor cells. They report on differentially regulated genes compared in proliferating and differentiating ENSs involved in pathways such as cell cycle, apoptosis, proliferation, or differentiation.

Besides the expected differences in cell cycle regulation they also reported on marked inactivation of the canonical Wnt-pathway after induction of differentiation.

In a second study on the enteric nervous system, K. Nothelfer et al. further extend the knowledge about the Wnt-Fzd-pathway in the ENS. They investigated the expression of the Wnt receptor frizzled-4 in the human ENS of small and large intestines and found a significant abundance of frizzled-4 in a subpopulation of enteric neuronal and glial cells. Moreover they observe a copositivity of frizzled-4 with neural progenitor markers nestin and p75ntr, implying a role of frizzled-4 during the development of the ENS.

On the reverse developmental pathway describing the reprogramming of somatic cells to induced pluripotent stem cells, a huge number of mechanisms and transcription factors are involved in the turn of maturity to pluripotency. Here, it was already reported that T-box transcription factors play distinct roles in the pluripotency network itself as well as during early steps of development. In this respect, M. Klingenstein et al. provide evidence that although Tbx3 is positively promoting iPS cell reprogramming, it still is not crucial for the generation or persistence of iPS cells. This information might fill a gap of knowledge concerning factors involved in both pluripotency and in early steps of cell fate determination, cell development, and adult function.

Signaling pathways and underlying factors represent the crucial set to upkeep stemness* in vivo *and* in vitro*. Nevertheless, during recent years it has become evident that these factors and their balance are also involved in early cell fate choice. In a summary on pluripotency factors, C. Weidgang et al. summarize current knowledge about the role of these factors not only in pluripotent conditions, but also during early cell fate determination, namely, the exit from pluripotency. Herein, a thorough and well-defined overview is provided as useful germ layer-related classifier of pluripotency factors.

Organ development occurs in a cell but also niche-dependent manner. Herein, the developing pancreas gives a bona fide example where pancreatic epithelial cells develop in branching morphogenesis while receiving important signaling input from the overlying mesenchyme. H. A. Russ and colleagues complemented a transgenic mouse model to label the pancreatic mesenchyme with subsequent proteomic analysis. Thereby, three potentially important factors for pancreatic development were identified, which the authors validated in directed differentiation efforts of human embryonic stem cells. Taken together, analyzing the proteome of supporting tissues in mouse models may aid to identify novel regulators of pancreatic differentiation of human pluripotent stems cells and thereby improve purity of currently available protocols.

Pancreatic development occurs in a stepwise and tightly regulated manner, a process strongly dependent on a transcription factor orchestra including Pdx1, Prrx1, or Sox9. M. Reichert and colleagues shed light on the role of such factors during initiation of pancreatic cancer. Herein, the authors outline the stepwise onset of various pancreatic cancer progenitor lesions and put the governing factors in both a developmental and cancerous context. In that sense they develop a model for developmental factors regulating acinar and duct cell transformation, to date the most critical and earliest event of pancreatic cancer onset.

Following this track, E. Hessmann and colleagues identified the Nfatc4-Sox9 axis as a critical driver of acinar cell plasticity and pancreatic cancer initiation. Oncogenic Kras requires secondary hits to drive premalignant lesions in the pancreas toward carcinogenesis. Herein, developmental pathways such as the epidermal growth factor signaling axis are sufficient to pass this premalignant border. The authors complemented acinar explant cultures with immunohistochemical methods and chromatin immunoprecipitation analysis to identify the fact that Nftac4 is activated by EGFR signaling and initiates acinar to ductal metaplasia by direct transcriptional activation of Sox9. Thereby, the authors provide additional evidence supporting a Janus face of Sox9 during development and cancer in the pancreas.

Finally, M. Quante et al. developed a novel 3D culture system to study cancerous and normal intestinal tissue in a niche-dependent context. The complementation of intestinal but also premalignant Barrett epithelium derived organoid cultures with various matrices and cocultured cell types enabled the group to study niche-dependent effects in the gastrointestinal tract. In turn, myofibroblasts but also neurons from the enteric nervous system boosted organoid growth and enhanced survival. The developed platform provides unique access to modulate and mimic cancerous growth* in vitro* and at the same time to study niche-dependence in self-renewing (cancer) stem cells.

Taken together, the current special issue provides a unique disquisition on embryonic and somatic stem cells in healthy but also diseased tissues and the impact of stem cell factors and niche-effects on their differentiation and self-renewal capacity. Again, the parallel lines between embryonic development and cancer onset, governed by similar factors in different contexts, become evident and disclose the importance to understand both similarities and discrepancies of these processes.

*Stefan Liebau* *Stefan Liebau* *Paul Gadue* *Paul Gadue* *Kodandaramireddy Nalapareddy* *Kodandaramireddy Nalapareddy* *Guido von Figura* *Guido von Figura* *Alexander Kleger* *Alexander Kleger*

